# Wroblewski’s Local Anesthetic

**Published:** 1897-12

**Authors:** 


### Wroblewski's Local Anæsthetic.

| | | |
|---|---|---|
| ℞ | Antipyrine - - - - - | 2 parts |
| | Cocaine - - - - - | 1 part |
| | Water - - - - - - | 10 parts |

Local application for tonsils and adenoids, before operation. —*Ann. Univ. Med. Soc.*, 1895.

